# Case Report: Central Nervous System Immune Reconstitution Inflammatory Syndrome Related to Bacterial Meningitis

**DOI:** 10.3389/fimmu.2021.585316

**Published:** 2021-03-29

**Authors:** Mengyan Hu, Sanxin Liu, Danli Lu, Yi Zhong, Dafan Yu, Wei Qiu, Zhengqi Lu, Bingjun Zhang

**Affiliations:** ^1^ Department of Neurology, Center for Mental and Neurological Disorders and Diseases, The Third Affiliated Hospital of Sun Yat-sen University, Guangzhou, China; ^2^ Department of Dermatology, Guangzhou Women And Children’s Medical Center, Guangzhou, China

**Keywords:** immune reconstitution inflammatory syndrome, bacterial meningitis, *Staphylococcus aureus*, pregnancy, case report

## Abstract

Central nervous system immune reconstitution inflammatory syndrome (CNS-IRIS) describes clinical characteristics that may be observed in previously immunocompromised patients during rapid restoration of immunity function in the presence of a pathogen. There have been no reports about CNS-IRIS related to bacterial meningitis so far. Here, we report a 24-year-old pregnant female patient with bacterial meningitis. Her clinical and neuroradiological condition worsened after induced labor despite great effective anti-infective therapy. CNS-IRIS was considered. Corticosteroids were administered, and the patient gradually recovered. We present the first case of CNS-IRIS associated with bacterial meningitis.

## Introduction

Infection of the central nervous system (CNS), usually presenting as meningitis or encephalitis, is linked to high mortality and morbidity rates throughout the world (1). During therapy of CNS infection, clinical deterioration may occur despite evidence of effective treatment. Known as CNS-immune reconstitution inflammatory syndrome (CNS-IRIS), this paradoxical heightened immune response was initially identified in human immunodeficiency virus (HIV)-infected patients following anti-infection therapy and reversal of immune deficiency with antiretroviral therapy (2). However, it is increasingly found in immunocompromised hosts (3). CNS-IRIS occurs in 9%–47% of persons with HIV infection and CNS opportunistic infection who start antiretroviral therapy and has a mortality rate of 13%–75% (4). These rates vary depending on the causative pathogen. Most common CNS-IRIS events develop in relation to cryptococcus, tuberculosis (TB), and John Cunningham (JC) virus, but several other viruses, mycobacteria, and fungi have been associated with CNS-IRIS.

However, to the best of our knowledge, there has not been report about CNS-IRIS related to bacterial meningitis so far. We describe an interesting case of CNS-IRIS in a pregnant patient with bacterial meningitis, who developed deteriorated neurologic symptoms as seen by brain imaging after induced labor and effective anti-infective therapy.

## Case Report

**Figure 1:**
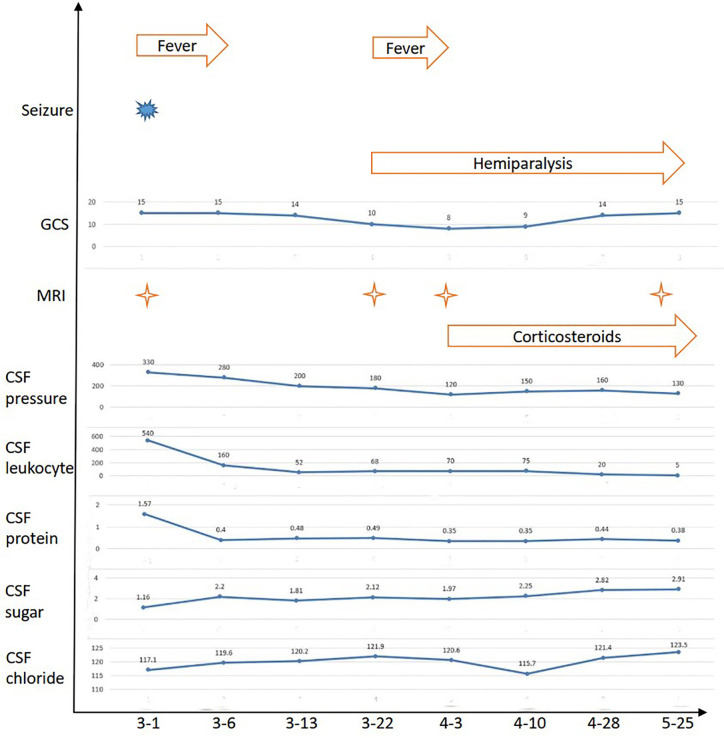
The clinical and CSF evolutions of the patient.

**Figure 2:**
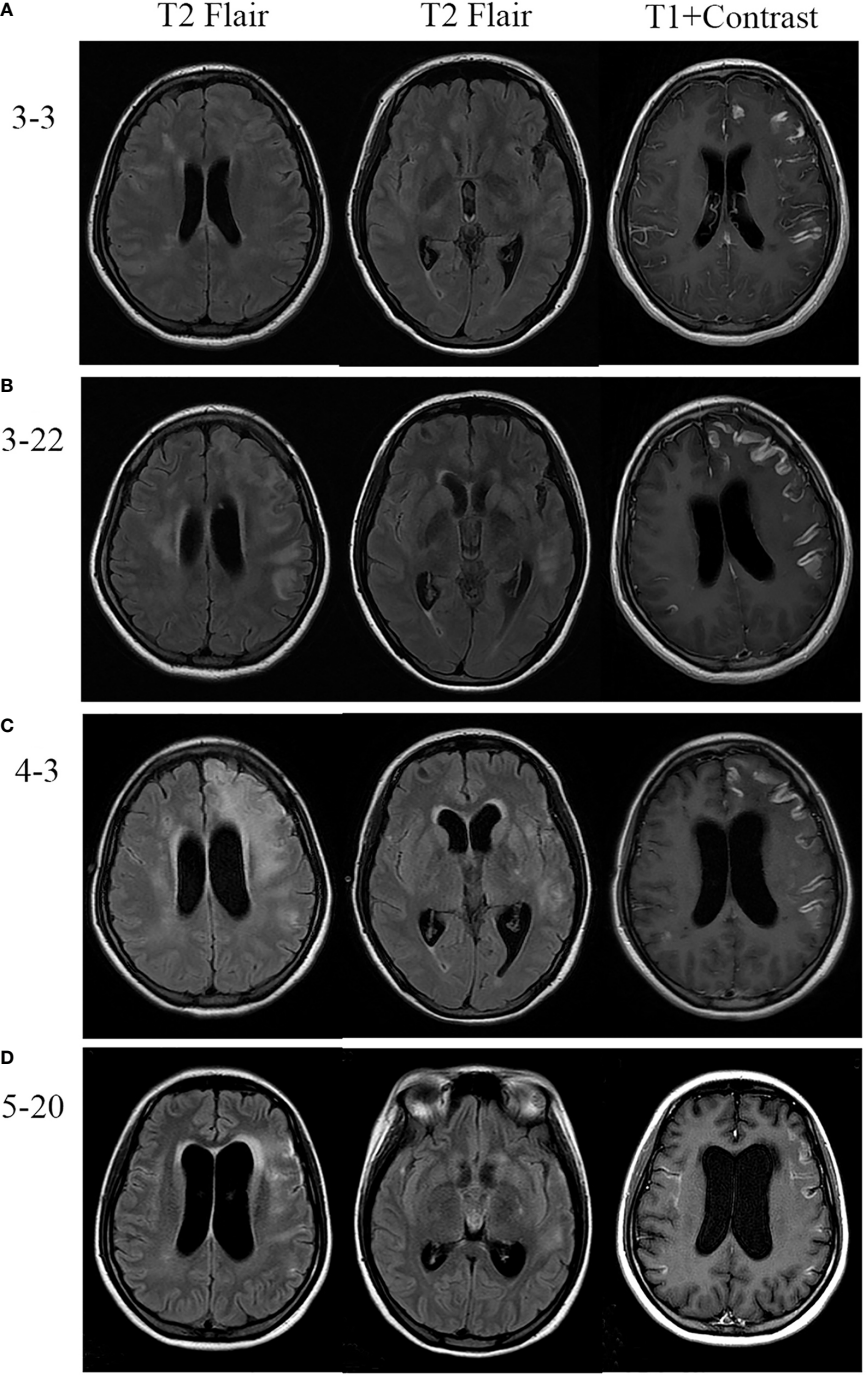
Brain MRI of the patient. **(A)** MRI revealed diffuse hyperintensities on fluid attenuated inversion recovery (FLAIR) imaging, accompanied by meningeal enhancement on the T1 contrast image. **(B, C)** Repeated MRI revealed that lesions were enlarged and meningeal enhancement was even more obvious after effective antibiotic therapy and induced labor. **(D)** Follow-up MRI showed marked improvement after corticosteroids treatment.

A 24-year-old pregnant female (G1P0) presented to the maternity ward of the local hospital, 12 weeks pregnant with a 14-day history of a mild headache and high fever for 2 days. After two generalized tonic-clonic seizure episodes, the female was transferred to our hospital. She had a 4-year history of hypothyroidism with irregular medication. The patient had no history of nasosinusitis, head trauma, or surgery. On admission, her vital signs revealed a temperature of 39.4°C, a blood pressure of 89/55 mmHg, a pulse of 120 beats/min, and a respiratory rate of 22 breaths/min. Physical examination revealed a lethargic patient with a Glasgow coma scale (GCS) score of 15/15. She had a normal physical examination except for nuchal rigidity and a positive Kernig’s sign. Blood tests showed that her leukocyte count was 22.7 × 10^9^/L (neutrophil 85.4%), sedimentation rate (ESR) 105 mm/H, C-reactive protein (CRP) 99.1 mg/L, and procalcitonin (PCT) 0.5 ng/ml. Lumbar puncture showed that her cerebrospinal fluid (CSF) pressure was 330 mmH_2_O, leukocyte count 540 × 10^6^/L (neutrophil 80.0%), protein 1.57 g/L, glucose 1.16 mmol/L, and chloride 117.1 mmol/L (**Figure 1**). The patient was diagnosed with bacterial meningitis, and treated immediately with empirical antibiotic therapy with meropenem (2.0 q8h intravenous drip) and vancomycin (1.0 q8h intravenous drip). Levetiracetam (1.0) was administered twice daily for antiepileptic treatment. Brain magnetic resonance imaging (MRI) revealed diffuse hyperintensities on fluid attenuated inversion recovery (FLAIR) imaging, accompanying by meningeal enhancement on a T1 contrast image (**Figure 2A**). Brain magnetic resonance angiography (MRA) imaging exhibited normal findings. Head CT and MRI showed no bone destructions and fistulas. Later, blood and CSF culture indicated staphylococcus aureus, which was sensitive to vancomycin and linezolid. The results were consistent with the analysis of metagenomics next-generation sequencing (mNGS). Thereafter, temperature was gradually decreased. There was no recurrence of seizures. Repeated blood and CSF results were significantly improved. Serum and CSF antibodies were negative for all antibodies against nerve cell-surface antigens and intracellular antigens. Autoantibodies and mycoplasma pneumoniae antibody were also negative. The T-SPOT.TB test was normal. There were no cardiac valve destructions and vegetations on the echocardiography exam. A Doppler ultrasound suggested fetal growth restriction.

The patient selected to abort the pregnancy because the fetus had growth restriction and had undergone medical interventions, including medications and imaging examinations, that had the potential to result in the teratogenic effect for the fetus. After 20 days of antibiotic therapy, the patient underwent a successful medication abortion. However, she developed weakness of the right limb, and fever again. She deteriorated with a fluctuating mental condition (GCS ranging from 10 to 12 of 15). Although brain MRA imaging were still normal, brain MRI revealed lesions were enlarged and meningeal enhancement was even more obvious (**Figure 2B**). Repeated multiple blood and CSF culture were negative for any bacteria. Linezolid (0.6 g bid intravenous drip) was used to as a substitute to vancomycin.

Unfortunately, the patient continued to deteriorate. She lapsed into a coma with a GCS 8 of 15. The repeated MRI was confirmed the progressive condition (**Figure 2C**). Antibodies related to autoimmune encephalitis were still negative in the serum and CSF. Therefore, CNS-IRIS was considered. The patient received high-dose intravenous methylprednisolone (20 mg/d/kg body weight, 5 days) followed by oral corticosteroids.

The neurologic abnormalities gradually improved, and follow-up MRI showed marked improvement (**Figure 2D**). She was discharged from hospital three months after her admission. Although weakness of the right limb continued, she was able to walk unaided.

## Discussion

This case demonstrated the unusual characteristics of bacterial meningitis in a pregnant patient. The clinical evolution of the patient was characterized by a paradoxical clinical condition and neuroradiological worsening after induced labor, despite the use of greatly effective anti-infective therapy. CNS-IRIS induced by bacterial meningitis was diagnosed and treated with corticosteroids. To the best of our knowledge, this is the first case of CNS-IRIS related to bacterial meningitis.

Bacterial meningitis is a medical emergency, and is associated with high mortality and disease burden. The epidemiology of community-acquired bacterial meningitis worldwide has changed in the past decades as a result of vaccination (5). The majority of bacterial meningitis cases in adults are due to streptococcus agalactiae and streptococcus pneumonia (6). Lymphatic dissemination, hematogenous dissemination, and direct extension from the adjacent areas are considered the possible routes of pathogenesis invasion into the CNS. Staphylococcus aureus meningitis is a relatively uncommon but serious CNS infectious disease. It is a nosocomial infection usually associated with neurosurgical procedures and head trauma, but spontaneous infections may occasionally appear in communities (7, 8). In the present study, staphylococcus aureus meningitis in our patient was a spontaneous community-acquired infection. Staphylococcus aureus usually invades the CNS *via* hematogenous spread on the basis of positive blood culture.

IRIS describes a series of symptoms and clinical characteristics that may be observed in previously immunocompromised patients during rapid restoration of immunity function in the presence of a pathogen or foreign antigen (2). However, there is no universally agreed-upon definition for IRIS. Immune deficiency is most frequently linked to HIV, a reversal of immunosuppressant states, and withdrawal of immunosuppressive drugs. IRIS can involve multiple organs, including the CNS, liver, lung, stomach, and intestine (9). The incidence of CNS-IRIS ranges widely according to CNS infection including TB, cryptococcus, and JC virus. An underlying mechanism of CNS-IRIS involves exposing large amounts of a foreign antigen to an immune system with improving ability which then responds and causes inflammation. Immune overactivation is caused by the hyper-responsiveness of the innate immune system as T lymphocyte function recovers (10). On brain biopsy or postmortem examinations, T lymphocytes and macrophages are found predominantly in the parenchyma and perivascular spaces (11). Brain MRI abnormalities, including new swelling with perilesional and perivascular edema, are often found in CNS-IRIS patients (12). The lesions might show contrast enhancement on MRI, due to local inflammation and breakdown of the blood–brain barrier (13, 14). Corticosteroids treatment is usually given to reduce the overactivation inflammatory response. In CNS-IRIS caused by TB and cryptococcus, corticosteroids treatment is recommended (4, 15, 16). However, use of corticosteroids in progressive multifocal leukoencephalopathy IRIS remains controversial (13). Furthermore, the dose and course of corticosteroids are still uncertain.

We believed that the patient met the definition of CNS-IRIS. First, the immunodeficient status was obvious during pregnancy in the patient. Second, immune function recovered rapidly after induced labor and effective antibiotic therapy. Third, deteriorated neurologic symptoms and brain imaging were found after treatment in the patient. Fourth, the deteriorated conditions were improved after use of corticosteroids. Fifth, the patient had no evidence of drug-resistant infection, bacterial superinfection, and drug allergy.

In conclusion, we present a rare case of a pregnant patient presenting with CNS-IRIS related to bacterial meningitis after effective antibiotic therapy and induced labor. Selection of the appropriate treatment regimen is challenging and is carefully considered in pregnant patients with severe infectious diseases.

## Data Availability Statement

All datasets generated for this study are included in the article.

## Ethics Statement

The studies involving human participants were reviewed and approved by the Medical Ethics Committee of the Third Affiliated Hospital of Sun Yat-sen University. Written informed consent to participate in this study was provided by the participants’ legal guardian/next of kin. Written informed consent was obtained from the participant’s next of kin for the publication of this case report, including any identifiable data or images included in this study.

## Author Contributions

MH, SL, DL, and BZ designed the research. YZ performed the experiments and analyzed the data. DY and BZ wrote the main manuscript text and prepared the figures. ZL, WQ, and BZ edited and revised the manuscript. All authors contributed to the article and approved the submitted version.

## Funding 

This study was supported by the National Natural Science Foundation of China (81971110, 81671178); the Guangdong Basic and Applied Basic Research Foundation (2020A1515010056); the Third Affiliated Hospital of Sun Yat-Sen University, Clinical Research Program (QHJH201907); and the Doctorial Starting Fund of Guangzhou Women and Children’s Medical Center (2018-293).

## Conflict of Interest

The authors declare that the research was conducted in the absence of any commercial or financial relationships that could be construed as a potential conflict of interest.
